# Optimally Distributed Kalman Filtering with Data-Driven Communication †

**DOI:** 10.3390/s18041034

**Published:** 2018-03-29

**Authors:** Katharina Dormann, Benjamin Noack, Uwe D. Hanebeck

**Affiliations:** 1 Robert Bosch GmbH, 71636 Ludwigsburg, Germany; katharina.dormann@gmx.de; 2 Intelligent Sensor-Actuator-Systems Laboratory (ISAS), Institute for Anthropomatics and Robotics, Karlsruhe Institute of Technology (KIT), 76131 Karlsruhe, Germany; benjamin.noack@ieee.org or uwe.hanebeck@ieee.org

**Keywords:** distributed Kalman Filtering, data-driven communication, distributed data fusion, sensor networks

## Abstract

For multisensor data fusion, distributed state estimation techniques that enable a local processing of sensor data are the means of choice in order to minimize storage and communication costs. In particular, a distributed implementation of the optimal Kalman filter has recently been developed. A significant disadvantage of this algorithm is that the fusion center needs access to each node so as to compute a consistent state estimate, which requires full communication each time an estimate is requested. In this article, different extensions of the optimally distributed Kalman filter are proposed that employ data-driven transmission schemes in order to reduce communication expenses. As a first relaxation of the full-rate communication scheme, it can be shown that each node only has to transmit every second time step without endangering consistency of the fusion result. Also, two data-driven algorithms are introduced that even allow for lower transmission rates, and bounds are derived to guarantee consistent fusion results. Simulations demonstrate that the data-driven distributed filtering schemes can outperform a centralized Kalman filter that requires each measurement to be sent to the center node.

## 1. Introduction

The efficient processing of sensor data is a central topic in a wide variety of research areas, which is underlined by advances in sensor technology and capabilities, e.g., for odor [1] and taste recognition [2] or by advances in visual information processing [3] as well as by applications in robotics [4] and sensor networks [5,6]. In particular to process data of multiple sensors, the well-known Kalman filter [7] has evolved into a key component of data fusion algorithms. Multisensor data can directly be transmitted to a data sink that employs a *centralized Kalman filter* to process all the accrued sensor readings. Such a simple filter design stands in stark contrast to the communication costs expended to transmit the data. The idea behind *distributed Kalman filter* implementations is to use local processing power to combine and condense sensor data locally so that the processing results can be transmitted more efficiently and less frequently. As the local processing results comprise the information from all past observations, the results can be sent to the data sink after arbitrarily many time steps without losing information from the past measurements. As compared to a centralized Kalman filter, distributed implementations not only offer the advantage that the communication load in the network can be reduced significantly but also increase robustness to packet losses and drops. The information in a lost package will automatically be handed in later through a subsequent data transmission.

Most distributed estimation architectures employ local Kalman filters at the sensor nodes. Although the local processing can then be deemed to be optimal in terms of the mean squared estimation error, fusion of the local estimates becomes a challenging task due to correlations between the estimates to be fused [8,9,10]. In particular, a naïve or too simplistic fusion method may render the fusion results inconsistent, i.e., the fusion method reports an estimation error that is too small to account for the actual error. In general, the correlations required to fuse the local estimates optimally are difficult to reconstruct [11,12]. In the case of unknown correlations, conservative fusion algorithms, like *Covariance Intersection* [13,14] or *Ellipsoidal Intersection* [15,16,17] and its further development *Inverse Covariance Intersection* [18,19], can be employed to obtain consistent fusion results. However, these algorithms often provide too conservative assessments of the actual estimation error.

An alternative approach to distributed state estimation has been pursued in [20,21,22,23]. Here, a distributed implementation of a single centralized Kalman filter has been derived. In doing so, the need to keep track of correlations or to derive conservative bounds can be circumvented. The main advantage of this approach is its optimality with respect to the mean squared estimation error; even optimal fusion algorithms, like the Bar-Shalom/Campo fusion rule, cannot achieve the same estimation quality [11]. Therefore, this approach constitutes an *optimally distributed Kalman filter* (ODKF). An important aspect of this filter is that the local estimates are neither consistent nor unbiased, nor does the error covariance matrix describe the actual estimation error. The local estimates have to be fused in the data sink to obtain a consistent estimate, which is optimal and equal to the result of a centralized Kalman filter. Beside this article, several extensions of this filter have been proposed—assumptions on the available information can be relaxed [24,25], it can be implemented in information form [26], or combinations with ellipsoidal state estimation are possible [27,28].

Although communication can take place at arbitrary time steps, a critical drawback of the ODKF is that the data sink requires access to all local estimates in order to compute an estimate, i.e., all nodes have to transmit their estimates to the center node. This limitation has the following two severe implications:

1. *All* nodes have to send their data at the *same* time, and
2. the central cannot infer any information about the state between the sending times.

Hence, the standard implementation of the ODKF implies transmissions of either all or none of the nodes. As a further consequence, full-rate communication of the nodes is required if the data sink needs an estimate at every time step. In this article, extensions of the ODKF are proposed that can operate under lower communication rates. This is achieved by introducing data-driven transmission strategies [29,30]. In particular, the local estimates can asynchronously be transmitted to the data sink. In order to guarantee consistency, bounds on the estimation errors are provided. These bounds are only required in situations when not every local estimate is available at the data sink; the optimal estimate as provided by a central Kalman filter is still obtained each time when all local estimate have been sent to the data sink.

As compared to the standard formulation of the ODKF, the advantage of the proposed extension is that the data sink can now compute an estimate of the state based on a subset of local estimates. This article continues the work in [31] by introducing an additional criterion for the data-driven transmission strategy, providing more details, and extending the evaluation and discussions.

The paper is structured as follows. Section 2 provides a description of the centralized and the optimally distributed Kalman filter as well as a problem formulation. In Section 3, the first extension is introduced which enables the data sink to treat missing estimates. In Section 4 and in Section 5, we describe the second and the third new distributed algorithm, respectively, which implement data-driven transmission schemes and allow for omitted estimates at the fusion center over multiple time steps. The results of an experimental evaluation are discussed in Section 6. Finally, the article is concluded in Section 7.

## 2. Centralized and Optimally Distributed Kalman Filtering

We consider a sensor network with *N* local sensor nodes and a central node, which serves as a data sink and computes an estimate on the state. The true state of the system at time step *k* is denoted by ${\underline{x}}_{k}$, which evolves according to the discrete-time linear dynamic system

$${\underline{x}}_{k+1}=A_{k}{\underline{x}}_{k}+{\underline{w}}_{k}\;,$$

where $A_{k}$ is the system matrix and ${\underline{w}}_{k}$ denotes the process noise, which is assumed to be zero-mean Gaussian noise, ${\underline{w}}_{k}∼N\left(\underline{0},C_{k}^{w}\right)$ with covariance matrix $C_{k}^{w}$. At each time step *k*, each sensor i∈{1,…,N} observes the state through the model

$${\underline{z}}_{k}^{i}=H_{k}^{i}{\underline{x}}_{k}+{\underline{v}}_{k}^{i}\;,$$

where $H_{k}^{i}$ is the measurement matrix and ${\underline{v}}_{k}^{i}$ the measurement noise, which is assumed to be Gaussian noise with zero mean, ${\underline{v}}_{k}∼N\left(\underline{0},C_{k}^{z,i}\right)$ with covariance matrix $C_{k}^{z,i}$. The measurement noise terms of different local sensors are assumed to be mutually uncorrelated. Also, the process and measurement noise terms for different time steps are uncorrelated.

For the *centralized Kalman filter* (CKF), each measurement is sent to the data sink, and fused by means of the formulas

(1) $$\begin{array}{cc} {\left(C_{k}^{e,c}\right)}^{-1}\;{\underline{\hat{x}}}_{k}^{e,c} & ={\left(C_{k}^{p,c}\right)}^{-1}{\underline{\hat{x}}}_{k}^{p,c}+\sum_{i=1}^{N}\;{\left(H_{k}^{i}\right)}^{T}\;\;{\left(C_{k}^{z,i}\right)}^{-1}\;\;{\underline{z}}_{k}^{i}\;,\\ {\left(C_{k}^{e,c}\right)}^{-1} & ={\left(C_{k}^{p,c}\right)}^{-1}+\sum_{i=1}^{N}{\left(H_{k}^{i}\right)}^{T}{\left(C_{k}^{z,i}\right)}^{-1}H_{k}^{i}\;. \end{array}$$

These equations correspond to the information form [32,33] of the measurement update of the standard Kalman filter. ${\underline{\hat{x}}}_{k}^{e,c}$ and $C_{k}^{e,c}$ denote the state estimate and the corresponding error covariance matrix after the fusion step, respectively. ${\underline{\hat{x}}}_{k}^{p,c}$ and $C_{k}^{p,c}$ denote the state estimate and the corresponding error covariance matrix computed by the prediction step of the Kalman filter. The prediction step is carried out at the center node by

(2) $$\begin{array}{cc} {\left(C_{k+1}^{p,c}\right)}^{-1}{\underline{\hat{x}}}_{k+1}^{p,c} & ={\left(C_{k+1}^{p,c}\right)}^{-1}A_{k}{\underline{\hat{x}}}_{k}^{e,c}\;,\\ {\left(C_{k+1}^{p,c}\right)}^{-1} & ={\left(A_{k}C_{k}^{e,c}A_{k}^{T}+C_{k}^{w}\right)}^{-1}\;, \end{array}$$

where these formulas are also given in the information form [26,32]. Since these equations correspond to the standard Kalman filter, the CKF is unbiased and optimal with respect to the minimum mean squared error. In particular, the computed error covariance matrix is equal to the actual estimation error, i.e.,

(3) $$C_{k}^{e,c}=E\left(\left({\underline{\hat{x}}}_{k}^{e,c}-{\underline{x}}_{k}\right){\left({\underline{\hat{x}}}_{k}^{e,c}-{\underline{x}}_{k}\right)}^{T}\right)\;.$$

In [20,21,22,23], a distributed implementation of the Kalman filter algorithm has been derived, which is algebraically equivalent to the centralized scheme, i.e., which is also unbiased, minimizes the mean squared error, and which fulfills (3). This is achieved by defining a local filtering scheme such that the fusion result is equal to results (1) and (2) of the CKF. We will describe this algorithm—the *optimally distributed Kalman filter* (ODKF)—in the following.

The local sensor nodes run modified versions of the Kalman filtering algorithm. They use so called *globalized* local states estimates and error covariance matrices (Although, strictly speaking, the local estimate and covariance matrix do not represent consistent estimates of the state, we denote them as local estimates). To initialize the ODKF, the local initial estimates and covariance matrices $\left({\underline{\hat{x}}}_{0}^{e,i},C_{0}^{e,i}\right)$ at the sensor nodes i∈{1,…,N}, which are usually identical, have to be replaced by the globalized estimates

$$\begin{array}{cc} {\underline{\bar{x}}}_{0}^{e,i} & =C_{0}^{e}\sum_{i=1}^{N}{\left(C_{0}^{e,i}\right)}^{-1}{\underline{\hat{x}}}_{0}^{e,i}\;,\\ {\bar{C}}_{0}^{e} & =N{\left(\sum_{i=1}^{N}{\left(C_{0}^{e,i}\right)}^{-1}\right)}^{-1}\;. \end{array}$$

Since the globalized error covariance matrix is equal for each sensor, the sensor index *i* is omitted. This equality also applies to all future time steps. The local prediction step is replaced by the globalized prediction equations

(4) $$\begin{array}{cc} {\underline{\bar{x}}}_{k+1}^{p,i} & =A_{k}{\underline{\bar{x}}}_{k}^{e,i}\;,\\ {\bar{C}}_{k+1}^{p} & =A_{k}{\bar{C}}_{k}^{e}A_{k}^{T}+NC_{k}^{w}\;. \end{array}$$

The local filtering steps are globalized by

(5) $$\begin{array}{cc} {\underline{\bar{x}}}_{k}^{e,i} & ={\bar{C}}_{k}^{e}\left({\left({\bar{C}}_{k}^{p}\right)}^{-1}{\underline{\bar{x}}}_{k}^{p,i}+{\left(H_{k}^{i}\right)}^{T}{\left(C_{k}^{z,i}\right)}^{-1}{\underline{z}}_{k}^{i}\right)\;,\\ {\bar{C}}_{k}^{e} & ={\left({\left({\bar{C}}_{k}^{p}\right)}^{-1}+\frac{1}{N}\sum_{i=1}^{N}{\left(H_{k}^{i}\right)}^{T}{\left(C_{k}^{z,i}\right)}^{-1}H_{k}^{i}\right)}^{-1}\;. \end{array}$$

The processing steps (4) and (5) are computed locally on each sensor node. Hence, measurements are not directly transmitted to the central node—instead, the local estimates are sent to the central node. As the globalized covariance matrix is equal for each node, it can also be computed in the central node.

In order to compute an estimate at an arbitrary time step *k*, the central node receives the globalized estimates $\left({\underline{\bar{x}}}_{k}^{e,i},{\bar{C}}_{k}^{e}\right)$ from each sensor *i* and fuses them according to

(6) $$\begin{array}{cc} {\left(C_{k}^{e,d}\right)}^{-1} & =\sum_{i=1}^{N}{\left({\bar{C}}_{k}^{e}\right)}^{-1}\;, \end{array}$$

(7) $$\begin{array}{cc} {\left(C_{k}^{e,d}\right)}^{-1}{\underline{\hat{x}}}_{k}^{e,d} & =\sum_{i=1}^{N}{\left({\bar{C}}_{k}^{e}\right)}^{-1}{\underline{\bar{x}}}_{k}^{e,i}\;. \end{array}$$

${\underline{\hat{x}}}_{k}^{e,d}$ denotes the state estimate after the fusion step at the data sink with corresponding error covariance matrix $C_{k}^{e,d}$. From Equations (6) and (7), we can easily accept that

$$\begin{array}{cc} C_{k}^{e,d} & =\frac{1}{N}{\bar{C}}_{k}^{e}\;,\\ {\underline{\hat{x}}}_{k}^{e,d} & =\frac{1}{N}\sum_{i=1}^{N}{\underline{\bar{x}}}_{k}^{e,i}\;. \end{array}$$

The same equations apply to the fusion of the predicted estimates and error covariance matrices in (4). In [20], it has been shown that the results are optimal, i.e.,

(8) $$\begin{array}{c} {\underline{\hat{x}}}_{k}^{e,d}={\underline{\hat{x}}}_{k}^{e,c}\;, \end{array}$$

(9) $$\begin{array}{c} C_{k}^{e,d}=C_{k}^{e,c}\;, \end{array}$$

where ${\underline{\hat{x}}}_{k}^{e,c}$ and $C_{k}^{e,c}$ are computed by the CKF, i.e., (1) and (2). The disadvantage of the centralized Kalman filter is that each sensor node has to transmit measurements of each time step to the central node. For the ODKF, we observe that communication in past time steps does not influence ${\underline{\hat{x}}}_{k}^{e,d}$ and $C_{k}^{e,d}$, i.e., the equalities hold independently of the past communication pattern in the distributed network. As a consequence of (8) and (9), we can see that

(10) $$C_{k}^{e,d}=E\left(\left({\underline{\hat{x}}}_{k}^{e,d}-{\underline{x}}_{k}\right){\left({\underline{\hat{x}}}_{k}^{e,d}-{\underline{x}}_{k}\right)}^{T}\right)\;$$

is equal to (3)—the ODKF is optimal.

A significant drawback of the ODKF implementation becomes apparent in the following situation. If only m<N sensors transmit their estimates to the fusion center at time step *k*, Equations (6) and (7) become

(11) $$\begin{array}{cc} {\left(C_{k}^{e,d}\right)}^{-1} & =\sum_{i=1}^{m}{\left({\bar{C}}_{k}^{e}\right)}^{-1}\;, \end{array}$$

$$\begin{array}{cc} {\left(C_{k}^{e,d}\right)}^{-1}{\underline{\hat{x}}}_{k}^{e,d} & =\sum_{i=1}^{m}{\left({\bar{C}}_{k}^{e}\right)}^{-1}{\underline{\bar{x}}}_{k}^{e,i}. \end{array}$$

The resulting ODKF estimate ${\underline{\hat{x}}}_{k}^{e,d}$ and error covariance matrix $C_{k}^{e,d}$ are different from the centralized estimate ${\underline{\hat{x}}}_{k}^{e,c}$ and the error covariance matrix $C_{k}^{e,c}$, which are

(12) $$\begin{array}{cc} {\left(C_{k}^{e,c}\right)}^{-1}{\underline{\hat{x}}}_{k}^{e,c}\; & =\;{\left(C_{k}^{p,c}\right)}^{-1}{\underline{\hat{x}}}_{k}^{p,c}+\sum_{i=1}^{m}{\left(H_{k}^{i}\right)}^{T}{\left(C_{k}^{z,i}\right)}^{-1}{\underline{z}}_{k}^{i}\;, \end{array}$$

(13) $$\begin{array}{cc} {\left(C_{k}^{e,c}\right)}^{-1} & ={\left(C_{k}^{p,c}\right)}^{-1}+\sum_{i=1}^{m}{\left(H_{k}^{i}\right)}^{T}{\left(C_{k}^{z,i}\right)}^{-1}H_{k}^{i}\;. \end{array}$$

In particular, we notice that the covariance matrix (13) differs from the ODKF covariance matrix in (11). A consequence of this mismatch is a possible bias in the fused estimate as discussed, e.g., in [24]—hence, the ODKF may provide inconsistent estimates in case of missing transmissions. This issue will be addressed in the following sections. Although the CKF does not suffer from inconsistency, (12) and (13) unveil a critical downside of the CKF: Missing measurements at time step *k* are lost for all future time steps. By contrast, the local estimates of the ODKF incorporate past measurements, which states the reason why the ODKF may outperform the CKF if we can solve the inconsistency problem of the ODKF.

In this section, the ODKF has been revisited; it provides the same results as the CKF but offers the advantage that transmissions can take place at arbitrary instants of time. However, the ODKF still requires that all nodes send their local estimates at the transmission times to compute (6) and (7). As a consequence, the data sink typically operates at a lower rate than the local nodes as it is idle between transmission times. In the following sections, extensions are provided that enable the nodes to transmit their local estimates asynchronously. The data sink can then operate at the same rate as the sensor nodes, i.e., it is able to provide an estimate at every time step *k*. By employing data-driven strategies, the communication rate of each node can be significantly lower than 1, where the value 1 corresponds to transmissions at every time step *k*.

In Section 3, we develop a consistent ODKF extension than can cope with situations where sensor nodes may send their estimates at every second time step. This algorithm still provides results equal to the CKF. With this algorithm, we are able to reduce the communication rate by half. Section 4 and Section 5 introduce a second and third algorithm that can even reach a lower communication rate by applying bounds on the missing pieces of information.

## 3. Distributed Kalman Filtering with Omitted Estimates

We consider the ODKF algorithm as described in the previous section. At time step *k*, only sensor nodes 1,…,m send their estimates to the fusion center, but the estimates of sensor nodes m+1,…,N are not. In this section, we assume that the data from the nodes m+1,…,N had been available in the fusion center at time step k−1. Thus, the fusion center can compute the predicted estimates ${\underline{\bar{x}}}_{k}^{p,m+1},\ldots ,{\underline{\bar{x}}}_{k}^{p,N}$ for time step *k* by using (4). In place of the ODKF fusion Equations (6) and (7), the fusion result is now computed by

(14) $$\begin{array}{cc} {\left(C_{k}^{e,d^{\prime }}\right)}^{-1} & =\sum_{i=1}^{N}{\left({\bar{C}}_{k}^{e}\right)}^{-1}-\sum_{i=m+1}^{N}{\left(H_{k}^{i}\right)}^{T}{\left(C_{k}^{z,i}\right)}^{-1}H_{k}^{i}\;, \end{array}$$

(15) $$\begin{array}{cc} {\left(C_{k}^{e,d^{\prime }}\right)}^{-1}{\underline{\hat{x}}}_{k}^{e,d^{\prime }} & =\sum_{i=1}^{m}{\left({\bar{C}}_{k}^{e}\right)}^{-1}{\underline{\bar{x}}}_{k}^{e,i}+\sum_{i=m+1}^{N}{\left({\bar{C}}_{k}^{p}\right)}^{-1}{\underline{\bar{x}}}_{k}^{p,i}\;\;\; \end{array}$$

The resulting estimate ${\underline{\hat{x}}}_{k}^{e,d}$ and the error covariance matrix $C_{k}^{e,d}$ are equal to the estimate ${\underline{\hat{x}}}_{k}^{e,c}$ and the error covariance matrix $C_{k}^{e,c}$ computed by a centralized Kalman filter according to (12) and (13). A proof for the equality is provided in Appendix A.

Since (14) and (15) are equivalent to the CKF, unbiasedness, optimality, and (10) are accordingly inherited from the CKF. We have generalized the original ODKF algorithm such that full-rate communication is not required anymore. The novel fusion algorithm merely requires that if a particular sensor does not communicate with the center at time *k*, it has sent its data at time k−1, i.e., each sensor has to communicate with the center at least every second time step. Hence, the required communication rate can be reduced by half to 0.5.

However, a higher communication rate—and thus, the incorporation of the information contained in additional measurements—will always result in a lower mean squared error (MSE). Thus, we have to deal with the trade-off between a low communication rate and a low MSE. Nevertheless, it is possible to achieve a smaller MSE while keeping the same communication rate by using a data-driven communication strategy and thus, scheduling the data according to the information contained. Valuable results have already been achieved using data-driven communication in distributed sensor networks [34,35,36,37,38,39,40,41,42]. The idea is that each local sensor evaluates the distance between the predicted estimate ${\underline{\bar{x}}}_{k}^{p,i}$ and the filtered estimate ${\underline{\bar{x}}}_{k}^{e,i}$. If the distance is large, the measurement ${\underline{z}}_{k}^{i}$ adds much new information to the prediction. Only in this case, the sensor should send its current estimate ${\underline{\bar{x}}}_{k}^{e,i}$ to the center node.

It is important to emphasize that the globalized parameters ${\underline{\bar{x}}}_{k}^{p,i}$ and ${\underline{\bar{x}}}_{k}^{e,i}$ are not unbiased estimates of the actual state. It can be shown [24] that, in contrast to the difference between the standard Kalman filter estimates, ${\underline{\hat{x}}}_{k}^{p,i}-{\underline{\hat{x}}}_{k}^{e,i}$, the difference ${\underline{\bar{x}}}_{k}^{p,i}-{\underline{\bar{x}}}_{k}^{e,i}$ is not zero on average, but may even diverge. Therefore, in order to evaluate the influence of a measurement ${\underline{z}}_{k}^{i}$, we study the difference between the predicted and updated estimates of the standard Kalman filter, which is related to the weighted difference between the measurement and the prediction, i.e.,

$$\begin{array}{c} {\underline{\hat{x}}}_{k}^{p,i}-{\underline{\hat{x}}}_{k}^{e,i}=K_{k}^{i}\left({\underline{z}}_{k}^{i}-H_{k}^{i}{\underline{\hat{x}}}_{k}^{p,i}\right)\;, \end{array}$$

where $K_{k}^{i}$ denotes the standard Kalman gain. For this purpose, the standard Kalman filtering algorithm has to run in parallel to the globalized version of the Kalman filter at each sensor node. The following data-driven communication strategy can be applied:

(16) $$\begin{array}{c} \;\mathrm{If}\;∥{\underline{\hat{x}}}_{k}^{p,i}-{\underline{\hat{x}}}_{k}^{e,i}∥\leq \alpha \\ \;\;\mathrm{do}\;\mathrm{not}\;\mathrm{send}\;\mathrm{estimate}\;\mathrm{to}\;\mathrm{the}\;\mathrm{fusion}\;\mathrm{center}\\ \;\mathrm{else}\;\\ \;\;\mathrm{send}\;\mathrm{estimate}\;\mathrm{to}\;\mathrm{the}\;\mathrm{fusion}\;\mathrm{center}, \end{array}$$

where α denotes a scalar parameter. We can achieve any communication rate in range [0.5,1] by varying the parameter α.

By using this communication strategy we can only evaluate the relevance of the measurement to the local estimate ${\underline{\hat{x}}}_{k}^{p,i}$, and not to the fused estimate ${\underline{\hat{x}}}_{k}^{p,d}$. Still, experiments (see Section 6) will show that by using the data-driven communication strategy instead of random communication, for a fixed communication rate an improvement of the MSE of the fused estimate ${\underline{\hat{x}}}_{k}^{e,d^{\prime }}$ can be achieved. However, a drawback of the algorithm is the assumption that if a particular sensor does not communicate with the center at time *k*, it has communicated at time k−1, i.e., each sensor communicates with the center at least every other time step. Thus, communication rates lower than 0.5 cannot be achieved. This will be addressed by the following extensions.

## 4. Data-Driven Distributed Kalman Filtering with Omitted Estimates over Multiple Time Steps–Version 1

If we want to achieve communication rates lower than 0.5 in the sensor network, we have to allow that a particular sensor does not send its estimates to the fusion center over multiple time steps. In this case, the fusion center has to perform multiple consecutive predictions. Let us assume that the last communication of sensor *i* with the center occurred at time k−l. The predicted estimate for time step *k* is computed as shown in the following scheme.

$$\begin{array}{c} {\underline{\bar{x}}}_{k-l}^{e,i}\overset{Prediction}{\to }{\underline{\bar{x}}}_{k-l+1}^{pp,i}\overset{Prediction}{\to }\ldots \overset{Prediction}{\to }{\underline{\bar{x}}}_{k}^{pp,i} \end{array}$$

Prediction refers to the application of Equation (4). Note that the predicted estimates are now marked with “pp” instead of “*p*” to emphasize that possibly multiple prediction steps were applied consecutively. In case that prediction has been performed only once, we have ${\underline{\bar{x}}}_{k}^{pp,i}={\underline{\bar{x}}}_{k}^{p,i}$.

In fusion Equation (15). the predictions ${\underline{\bar{x}}}_{k}^{pp,i}$ are now used for the missing local estimates of the nodes i=m+1,…,N, i.e.,

(17) $${\left(C_{k}^{e,d^{\prime }}\right)}^{-1}{\underline{\hat{x}}}_{k}^{e,d^{\prime \prime }}=\sum_{i=1}^{m}{\left({\bar{C}}_{k}^{e}\right)}^{-1}{\underline{\bar{x}}}_{k}^{e,i}+\sum_{i=m+1}^{N}{\left({\bar{C}}_{k}^{p}\right)}^{-1}{\underline{\bar{x}}}_{k}^{pp,i}\;.$$

The new estimate ${\underline{\hat{x}}}_{k}^{e,d^{\prime \prime }}$ can be expressed in terms of the estimate ${\underline{\hat{x}}}_{k}^{e,d^{\prime }}$ from (15) as follows:

$$\begin{array}{c} {\underline{\hat{x}}}_{k}^{e,d^{\prime \prime }}=C_{k}^{e,d^{\prime }}\left(\sum_{i=1}^{m}{\left({\bar{C}}_{k}^{e}\right)}^{-1}{\underline{\bar{x}}}_{k}^{e,i}+\sum_{i=m+1}^{N}{\left({\bar{C}}_{k}^{p}\right)}^{-1}{\underline{\bar{x}}}_{k}^{pp,i}\right)\\ =C_{k}^{e,d^{\prime }}({\left(C_{k}^{e,d^{\prime }}\right)}^{-1}{\underline{\hat{x}}}_{k}^{e,d^{\prime }}-\sum_{i=m+1}^{N}{\left({\bar{C}}_{k}^{p}\right)}^{-1}{\underline{\bar{x}}}_{k}^{p,i}\\ +\sum_{i=m+1}^{N}{\left({\bar{C}}_{k}^{p}\right)}^{-1}{\underline{\bar{x}}}_{k}^{pp,i})\\ ={\underline{\hat{x}}}_{k}^{e,d^{\prime }}-C_{k}^{e,d^{\prime }}{\left({\bar{C}}_{k}^{p}\right)}^{-1}\sum_{i=m+1}^{N}\left({\underline{\bar{x}}}_{k}^{pp,i}-{\underline{\bar{x}}}_{k}^{p,i}\right)\;. \end{array}$$

For the yet to be defined triggering criterion, we consider the distance

(18) $$d_{k}^{i}:={\underline{\bar{x}}}_{k}^{pp,i}-{\underline{\bar{x}}}_{k}^{p,i}\;.$$

The expected estimation error covariance matrix is then given by

$$\begin{array}{c} E\left(\left({\underline{x}}_{k}-{\underline{\hat{x}}}_{k}^{e,d^{\prime \prime }}\right){\left({\underline{x}}_{k}-{\underline{\hat{x}}}_{k}^{e,d^{\prime \prime }}\right)}^{T}\right)\\ =E(\left({\underline{x}}_{k}-{\underline{\hat{x}}}_{k}^{e,d^{\prime }}-C_{k}^{e,d^{\prime }}{\left({\bar{C}}_{k}^{p}\right)}^{-1}\sum_{i=m+1}^{N}d_{k}^{i}\right)\\ {\left({\underline{x}}_{k}-{\underline{\hat{x}}}_{k}^{e,d^{\prime }}-C_{k}^{e,d^{\prime }}{\left({\bar{C}}_{k}^{p}\right)}^{-1}\sum_{i=m+1}^{N}d_{k}^{i}\right)}^{T})\;. \end{array}$$

Obviously, the expected estimation error cannot be computed exactly at the fusion center, since the difference $d_{k}^{i}$ is not available. Nevertheless, it is possible to obtain an upper bound on the estimation error, if we alter the communication test (16). We ensure that in case of communication the matrix $d_{k}^{i}{\left(d_{k}^{i}\right)}^{T}$ is bounded by B. The communication strategy becomes

(19) $$\begin{array}{c} \mathrm{If}\;∥{\underline{\hat{x}}}_{k}^{pp,i}-{\underline{\hat{x}}}_{k}^{e,i}∥\leq \alpha \;\mathrm{and}\;d_{k}^{i}{\left(d_{k}^{i}\right)}^{T}\leq B\\ \;\;\mathrm{do}\;\mathrm{not}\;\mathrm{send}\;\mathrm{estimate}\;\mathrm{to}\;\mathrm{the}\;\mathrm{fusion}\;\mathrm{center}\;\\ \;\mathrm{else}\;\\ \;\;\mathrm{send}\;\mathrm{estimate}\;\mathrm{to}\;\mathrm{the}\;\mathrm{fusion}\;\mathrm{center}, \end{array}$$

where ${\underline{\hat{x}}}_{k}^{pp,i}$ denotes the estimate which has been computed by applying the Kalman filtering prediction step multiple times and B denotes a user-defined symmetric positive definite matrix. For any square matrices X and Y, X≤Y denotes that Y−X is positive semi-definite.

For the bound, the relationship

(20) $$\begin{array}{c} d_{k}^{i}{\left(d_{k}^{i}\right)}^{T}\leq B\Leftrightarrow {\left(d_{k}^{i}\right)}^{T}B^{-1}d_{k}^{i}\leq 1 \end{array}$$

holds. Thus, the communication criterion $d_{k}^{i}{\left(d_{k}^{i}\right)}^{T}\leq B$ can be replaced by ${\left(d_{k}^{i}\right)}^{T}B^{-1}d_{k}^{i}\leq 1$, which reduces the computational cost. Assuming that $d_{k}^{i}$ is an *n*-dimensional vector and B an n×n-matrix, the cost for computing the product $d_{k}^{i}{\left(d_{k}^{i}\right)}^{T}$ is in $O(n^{2})$. The computation of the difference $B-d_{k}^{i}{\left(d_{k}^{i}\right)}^{T}$ is also in $O(n^{2})$. The computational cost for computing the eigenvalues of the n×n-matrix $B-d_{k}^{i}{\left(d_{k}^{i}\right)}^{T}$ is in $O(n^{3})$. All in all, the computational cost for the decision if $d_{k}^{i}{\left(d_{k}^{i}\right)}^{T}\leq B$ is in $O(n^{3}+n^{2})$. Since the inverse matrix $B^{-1}$ has to be computed only once beforehand, the cost for computing the product ${\left(d_{k}^{i}\right)}^{T}B^{-1}d_{k}^{i}$ is in $O(n^{2})$. Therefore, testing if ${\left(d_{k}^{i}\right)}^{T}B^{-1}d_{k}^{i}\leq 1$ only requires $O(n^{2})$ computations. Since $∥{\underline{\hat{x}}}_{k}^{pp,i}-{\underline{\hat{x}}}_{k}^{e,i}∥\leq \alpha$ only requires the computation of the Euclidean norm, which is in O(n), the computational cost of the communication test (19) is in $O(n^{2})$. The employed relationship (20) is proven in Appendix B.

As in the previous communication strategy, we have to take into consideration that the globalized estimates ${\underline{\bar{x}}}_{k}^{pp,i}$ and ${\underline{\bar{x}}}_{k}^{p,i}$ are *biased* and thus, the difference $d_{k}^{i}$ may diverge. As a consequence, the matrix $d_{k}^{i}{\left(d_{k}^{i}\right)}^{T}$ may also diverge. In contrast to the previous fusion algorithm, using the standard Kalman filtering estimates ${\underline{\hat{x}}}_{k}^{pp,i}$, ${\underline{\hat{x}}}_{k}^{p,i}$ is not a reasonable solution, since the communication strategy is used to get an upper bound on $d_{k}^{i}$. An alternative possibility to avoid the divergence of the difference is to *debias* the local globalized estimates. A strategy to debias the estimates using *debiasing matrices* has been proposed in [24,25]. In each prediction and filtering step, each local node computes a new debiasing matrix. This matrix is initialized by $\Delta_{0}^{p,i}=I$. In the filtering step, the debiasing matrix is computed by

$$\begin{array}{c} \Delta_{k}^{e,i}={\bar{C}}_{k}^{e,i}{\left({\bar{C}}_{k}^{p,i}\right)}^{-1}\Delta_{k}^{p,i}+{\bar{C}}_{k}^{e,i}{\left(H_{k}^{i}\right)}^{T}{\left(C_{k}^{z,i}\right)}^{-1}H_{k}^{i}\;. \end{array}$$

In the prediction step, the debiasing matrix is computed by

(21) $$\begin{array}{c} \Delta_{k}^{p,i}=A_{k}\Delta_{k}^{e,i}A_{k}^{-1}\;. \end{array}$$

$\Delta_{k}^{pp,i}$ is computed by applying Equation (21) multiple times, until the next communication with the center node occurs. By multiplying the inverse of the debiasing matrix with the globalized estimates, we can debias the estimates [24,25], i.e.,

$$\begin{array}{cc} E\left({\left(\Delta_{k}^{e,i}\right)}^{-1}{\underline{\bar{x}}}_{k}^{e,i}\right) & =E\left({\underline{x}}_{k}\right), \end{array}$$

$$\begin{array}{cc} E\left({\left(\Delta_{k}^{p,i}\right)}^{-1}{\underline{\bar{x}}}_{k}^{p,i}\right) & =E\left({\underline{x}}_{k}\right)\;. \end{array}$$

It can be easily shown that the same applies to the predicted estimate over multiple time steps, i.e.,

$$\begin{array}{c} E\left({\left(\Delta_{k}^{pp,i}\right)}^{-1}{\underline{\bar{x}}}_{k}^{pp,i}\right)=E\left({\underline{x}}_{k+n}\right)\;. \end{array}$$

We new define

$$\begin{array}{c} {\underline{\tilde{x}}}_{k}^{pp,i}:=\Delta_{k}^{p,i}{\left(\Delta_{k}^{pp,i}\right)}^{-1}{\underline{\bar{x}}}_{k}^{pp,i} \end{array}$$

and then have

$$\begin{array}{c} E\left({\underline{\tilde{x}}}_{k}^{pp,i}-{\underline{\bar{x}}}_{k}^{p,i}\right)=E\left(\Delta_{k}^{p,i}{\left(\Delta_{k}^{pp,i}\right)}^{-1}{\underline{\bar{x}}}_{k}^{pp,i}-{\underline{\bar{x}}}_{k}^{p,i}\right)\\ =\Delta_{k}^{p,i}E\left({\left(\Delta_{k}^{pp,i}\right)}^{-1}{\underline{\bar{x}}}_{k}^{pp,i}-{\left(\Delta_{k}^{p,i}\right)}^{-1}{\underline{\bar{x}}}_{k}^{p,i}\right)\\ =\Delta_{k}^{p,i}E\left({\underline{x}}_{k+n}-{\underline{x}}_{k}\right)\;. \end{array}$$

Thus, in general the difference ${\underline{\tilde{x}}}_{k}^{pp,i}-{\underline{\bar{x}}}_{k}^{p,i}$ does not diverge. With this result, we adapt (18), and use

$${\tilde{d}}_{k}^{i}:={\underline{\tilde{x}}}_{k}^{pp,i}-{\underline{\bar{x}}}_{k}^{p,i}$$

to replace the second inequality in (19) by

$${\tilde{d}}_{k}^{i}{\left({\tilde{d}}_{k}^{i}\right)}^{T}\leq B\;.$$

We can now define the new fusion equations as

$$\begin{array}{c} C_{k}^{e,d^{\prime \prime }}=C_{k}^{e,d^{\prime }}\\ +{\left(N-m-l\right)}^{2}C_{k}^{e,d^{\prime }}{\left({\bar{C}}_{k}^{p}\right)}^{-1}B{\left({\bar{C}}_{k}^{p}\right)}^{-1}{\left(C_{k}^{e,d^{\prime }}\right)}^{T}\\ {\underline{\hat{x}}}_{k}^{e,d^{\prime \prime }}=C_{k}^{e,d^{\prime }}\left(\sum_{i=1}^{m}{\left({\bar{C}}_{k}^{e}\right)}^{-1}{\underline{\bar{x}}}_{k}^{e,i}+\sum_{i=m+1}^{N}{\left({\bar{C}}_{k}^{p}\right)}^{-1}{\underline{\tilde{x}}}_{k}^{pp,i}\right)\;, \end{array}$$

where *m* is the number of sensors which communicate with the center at time *k* and *l* is the number of sensors which do not communicate with the center at time *k*, but for which ${\underline{\tilde{x}}}_{k}^{pp,i}-{\underline{\bar{x}}}_{k}^{p,i}=0$ hold. Note that the fusion formulas are equal to (14) and (15) for N=m+l, i.e., for the case that each sensor sends its estimate to the center at least every other time step. The resulting estimate is consistent, i.e.,

(22) $$E\left(\left({\underline{x}}_{k}-{\underline{\hat{x}}}_{k}^{e,d^{\prime \prime }}\right){\left({\underline{x}}_{k}-{\underline{\hat{x}}}_{k}^{e,d^{\prime \prime }}\right)}^{T}\right)\leq C_{k}^{e,d^{\prime \prime }}\;.$$

A proof for the consistency condition (22) is provided in Appendix C.

The drawback of the presented algorithm is that it needs two parameters B and α to perform the communication test. Both parameters influence the communication rate. Thus, it is difficult to find the parameters which ensure the desired balance between a small communication rate and a small estimation error. Experiments using the particular dynamic system are needed to find the best combination of both parameters. Thus, we will now present another algorithm which only uses one parameter B for the communication strategy.

## 5. Data-Driven Distributed Kalman Filtering with Omitted Estimates over Multiple Time Steps–Version 2

For the second data-driven algorithm, fusion Equation (15) is now generalized by

$${\left(C_{k}^{e,d^{\prime }}\right)}^{-1}{\underline{\hat{x}}}_{k}^{e,d^{\prime \prime }}=\sum_{i=1}^{m}{\left({\bar{C}}_{k}^{e}\right)}^{-1}{\underline{\bar{x}}}_{k}^{e,i}+\sum_{i=m+1}^{N}{\left({\bar{C}}_{k}^{e}\right)}^{-1}{\underline{\bar{x}}}_{k}^{pp,i}\;.$$

The difference to the previous Equation (17) is the covariance matrix ${\left({\bar{C}}_{k}^{e}\right)}^{-1}$ in the second sum. The new estimate ${\underline{\hat{x}}}_{k}^{e,d^{\prime \prime }}$ can be expressed in terms of the estimate ${\underline{\hat{x}}}_{k}^{e,d^{\prime }}$ from (15) as follows:

$$\begin{array}{c} {\underline{\hat{x}}}_{k}^{e,d^{\prime \prime }}=C_{k}^{e,d^{\prime }}\left(\sum_{i=1}^{m}{\left({\bar{C}}_{k}^{e}\right)}^{-1}{\underline{\bar{x}}}_{k}^{e,i}+\sum_{i=m+1}^{N}{\left({\bar{C}}_{k}^{e}\right)}^{-1}{\underline{\bar{x}}}_{k}^{pp,i}\right)\\ =C_{k}^{e,d^{\prime }}(\sum_{i=1}^{N}{\left({\bar{C}}_{k}^{e}\right)}^{-1}{\underline{\bar{x}}}_{k}^{e,i}-\sum_{i=m+1}^{N}{\left({\bar{C}}_{k}^{e}\right)}^{-1}{\underline{\bar{x}}}_{k}^{e,i}\\ +\sum_{i=m+1}^{N}{\left({\bar{C}}_{k}^{e}\right)}^{-1}{\underline{\bar{x}}}_{k}^{pp,i})\\ =C_{k}^{e,d^{\prime }}\sum_{i=1}^{N}{\left({\bar{C}}_{k}^{e}\right)}^{-1}{\underline{\bar{x}}}_{k}^{e,i}+C_{k}^{e,d^{\prime }}{\left({\bar{C}}_{k}^{e}\right)}^{-1}\sum_{i=m+1}^{N}\left({\underline{\bar{x}}}_{k}^{pp,i}-{\underline{\bar{x}}}_{k}^{e,i}\right)\\ ={\underline{\hat{x}}}_{k}^{e,d^{\prime }}+\frac{1}{N}\sum_{i=m+1}^{N}\left({\underline{\bar{x}}}_{k}^{pp,i}-{\underline{\bar{x}}}_{k}^{e,i}\right)\;. \end{array}$$

In order to define a communication strategy, we consider the difference

$$\begin{array}{c} d_{k}^{i}={\underline{\bar{x}}}_{k}^{pp,i}-{\underline{\bar{x}}}_{k}^{e,i}, \end{array}$$

which is compared against the matrix B by

$$\begin{array}{c} \mathrm{If}\;d_{k}^{i}{\left(d_{k}^{i}\right)}^{T}\leq B\\ \;\;\mathrm{do}\;\mathrm{not}\;\mathrm{send}\;\mathrm{estimate}\;\mathrm{to}\;\mathrm{the}\;\mathrm{fusion}\;\mathrm{center}\;\\ \;\mathrm{else}\;\\ \;\;\mathrm{send}\;\mathrm{estimate}\;\mathrm{to}\;\mathrm{the}\;\mathrm{fusion}\;\mathrm{center}. \end{array}$$

B denotes a user-defined symmetric positive definite matrix. This time we do not need the Euclidean distance $∥{\underline{\hat{x}}}_{k}^{pp,i}-{\underline{\hat{x}}}_{k}^{e,i}∥\leq \alpha$ since the distance between predicted and filtered estimate is included in $d_{k}^{i}$. We can now define the new fusion equations as

$$\begin{array}{c} C_{k}^{e,d^{\prime \prime }}=C_{k}^{e,d^{\prime }}+\frac{1}{N^{2}}{\left(N-m\right)}^{2}B\\ {\underline{\hat{x}}}_{k}^{e,d^{\prime \prime }}=C_{k}^{e,d^{\prime }}\left(\sum_{i=1}^{m}{\left({\bar{C}}_{k}^{e}\right)}^{-1}{\underline{\bar{x}}}_{k}^{e,i}+\sum_{i=m+1}^{N}{\left({\bar{C}}_{k}^{e}\right)}^{-1}{\underline{\bar{x}}}_{k}^{pp,i}\right)\;, \end{array}$$

where *m* is the number of sensors which communicate with the center at time *k*. With the same arguments as in Section 4 it can be shown that the resulting estimate is consistent, i.e.,

$$E\left(\left({\underline{x}}_{k}-{\underline{\hat{x}}}_{k}^{e,d^{\prime \prime }}\right){\left({\underline{x}}_{k}-{\underline{\hat{x}}}_{k}^{e,d^{\prime \prime }}\right)}^{T}\right)\leq C_{k}^{e,d^{\prime \prime }}\;.$$

In the experimental evaluation of the algorithms we will see that although this version of fusing the estimates has the advantage that only one parameter has to be chosen, the estimate of the fused error covariance matrix is not as good as as in the previous version.

## 6. Simulations and Evaluation

We apply the CKF algorithm as well as the three data-driven ODKF algorithms to a single-target tracking problem. The system state ${\underline{x}}_{k}$ is a six-dimensional vector with two dimensions for the position, two dimensions for the velocity, and two dimensions for the acceleration. A near-constant acceleration model is used. The system matrix is given by

$$\begin{array}{cc} A_{k} & =\left(\begin{array}{cccccc} 1 & \Delta & \Delta^{2}/2 & 0 & 0 & 0\\ 0 & 1 & \Delta & 0 & 0 & 0\\ 0 & 0 & 1 & 0 & 0 & 0\\ 0 & 0 & 0 & 1 & \Delta & \Delta^{2}/2\\ 0 & 0 & 0 & 0 & 1 & \Delta \\ 0 & 0 & 0 & 0 & 0 & 1 \end{array}\right)\;. \end{array}$$

with the sampling interval Δ=0.25 s. The process noise covariance matrix is given by

$$\begin{array}{cc} C_{k}^{w} & =\left(\begin{array}{cccccc} \Delta^{5}/20 & \Delta^{4}/8 & \Delta^{3}/6 & 0 & 0 & 0\\ \Delta^{4}/8 & \Delta^{3}/3 & \Delta^{2}/2 & 0 & 0 & 0\\ \Delta^{3}/6 & \Delta^{2}/2 & \Delta & 0 & 0 & 0\\ 0 & 0 & 0 & \Delta^{5}/20 & \Delta^{4}/8 & \Delta^{3}/6\\ 0 & 0 & 0 & \Delta^{4}/8 & \Delta^{3}/3 & \Delta^{2}/2\\ 0 & 0 & 0 & \Delta^{3}/6 & \Delta^{2}/2 & \Delta \end{array}\right)\;. \end{array}$$

We have a sensor network consisting of six sensor nodes and one fusion node. Two sensors measure the position, two sensors measure the velocity and two sensors measure the acceleration. The measurement noise covariance matrices are given by

$$\begin{array}{cc} C_{k}^{z,i} & =\left(\begin{array}{cc} 1 & 0\\ 0 & 1 \end{array}\right)\;\mathrm{for}\;i\in 1,\ldots ,6\;. \end{array}$$

Monte Carlo simulations with 500 independent runs over 100 time steps are performed. Since $\left(A_{k},C_{k}^{w}\right)$ is stable and $\left(A_{k},H_{k}\right)$ is detectable the error covariance matrix and the MSE converge to a unique values [43]. Based on the simulation, actual error covariance matrices and MSE values are computed for each algorithm. Monte Carlo simulations are performed for different average communication rates for each of the three algorithms. For the CKF, communication is performed randomly, but with different average rates. Note that only current measurements are communicated. If the measurement ${\underline{z}}_{k}^{i}$ is not sent to the fusion center at time *k*, the information will not be available at the center at any future time.

The first ODKF algorithm (Algorithm 1) from Section 3 is performed with random communication as well as with data-driven communication. In the latter case, the parameter α is varied to achieve different rates. The second algorithm (Algorithm 2) from Section 4 and the third algorithm (Algorithm 3) from Section 5 are performed with data-driven communication. For Algorithm 2 both parameters α and B are varied, for Algorithm 3 the parameter B is varied. The compared methods are:

**Algorithm** **1:** ODKF algorithm from Section 3 with random and data-driven communication.
**Algorithm** **2:** ODKF algorithm from Section 4 with data-driven communication and parameters α, B.
**Algorithm** **3:** ODKF algorithm from Section 5 with data-driven communication and parameter B.

**Figure 1:**
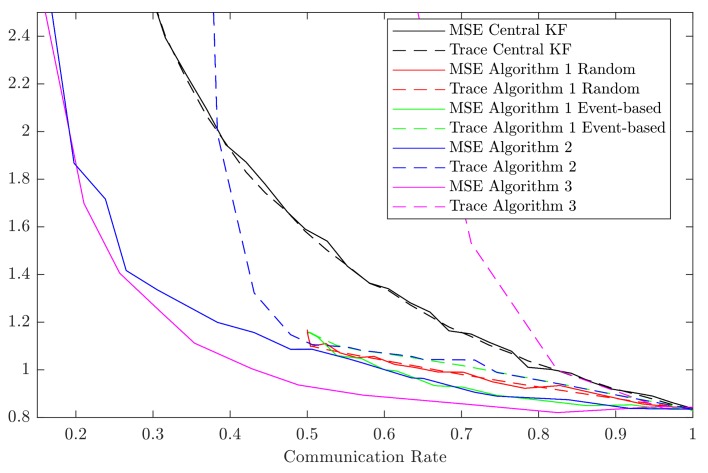
MSEs and traces of the error covariance matrices are plotted relative to the communication rate. Each communication rate corresponds to one Monte Carlo simulation with 500 runs over 100 time steps. MSEs are shown as solid lines, traces are shown as dashed lines.

The simulation results are shown in Figure 1. The MSEs and the traces of the error covariance matrices are depicted relative to the average communication rate in the network. Since for Algorithm 2 different parameter combinations lead to different results, we have only included the results with the smallest error covariance matrices in the plot.

Only for the centralized Kalman filter and for the Algorithm 2 and 3, communication rates lower than 0.5 are given. We can observe that for Algorithm 1 data-driven communication leads to an improved estimate compared to random communication. However, it also leads to a larger trace of the error covariance matrix and thus, to a larger uncertainty reported with the estimate.

We also can observe that for communication rates in range [0.5,1] the results of Algorithms 2 and 3 with data-driven communication are almost equal. This can be explained by the fact that Algorithm 2 extends Algorithm 3 share a common triggering criterion, and the fusion formulas for both algorithms are equal if each sensor communicates with the center at least every other time step.

Figure 1 shows that for each of the algorithms the MSE is always smaller than or equal to the trace of the error covariance matrix. This illustrates that the estimators provide consistent results. The traces are good estimates of the MSEs except for low communication rates in Algorithm 3 and very low communication rates in Algorithm 2. Thus, the trace of the error covariance matrices—the uncertainty reported by the estimators—is not significantly larger than the actual uncertainty in most of the cases. Each of the distributed fusion algorithms performs better in terms of the MSE as compared to the centralized algorithm. This can be explained by the fact that in the distributed network the fused estimates contain the information of all past measurements, while in the centralized network only the current measurements are fused.

## 7. Conclusions

In this article, the optimally distributed Kalman filter (ODKF) has been extended by data-driven communication strategies in order to bypass the need for full communication that is usually required by the ODKF to compute an estimate. Since the ODKF may provide inconsistent results if data transmissions are omitted, the missing estimates are replaced by predictions from previous time steps and consistent bounds on the error covariance matrix are computed. The first proposed technique allows for communication rates in the range [0.5,1] while the second and the third algorithm allow for any communication rate in range [0,1]. In a centralized Kalman filter (CKF), where measurements are directly sent to the central node, missing or lost transmissions to the center node need to be repeated in order to avoid loss of measurement data. In this regard, the proposed extensions of the ODKF can significantly outperform the CKF: The local estimates of each sensor node comprise the entire history of local measurements and hence, suspended transmissions do not lead to a loss of information in the network.

## Appendix A

We will now show that the resulting estimate ${\underline{\hat{x}}}_{k}^{e,d}$ and the error covariance matrix $C_{k}^{e,d}$ are equal to the estimate ${\underline{\hat{x}}}_{k}^{e,c}$ and the error covariance matrix $C_{k}^{e,c}$ computed by the CKF according to (12) and (13). *Proof:* $C_{k}^{e,d^{\prime }}=C_{k}^{e,c}$ holds due to

$$\begin{array}{c} {\left(C_{k}^{e,d^{\prime }}\right)}^{-1}=\sum_{i=1}^{N}{\left({\bar{C}}_{k}^{e}\right)}^{-1}\;-\;\sum_{i=m+1}^{N}{\left(H_{k}^{i}\right)}^{T}{\left(C_{k}^{z,i}\right)}^{-1}H_{k}^{i}\\ =N\left({\left({\bar{C}}_{k}^{e}\right)}^{-1}-\frac{1}{N}\sum_{i=m+1}^{N}{\left(H_{k}^{i}\right)}^{T}{\left(C_{k}^{z,i}\right)}^{-1}H_{k}^{i}\right)\\ =N({\left({\bar{C}}_{k}^{p}\right)}^{-1}+\frac{1}{N}\sum_{i=1}^{N}{\left(H_{k}^{i}\right)}^{T}{\left(C_{k}^{z,i}\right)}^{-1}H_{k}^{i}\\ -\frac{1}{N}\sum_{i=m+1}^{N}{\left(H_{k}^{i}\right)}^{T}{\left(C_{k}^{z,i}\right)}^{-1}H_{k}^{i})\\ =N\left({\left({\bar{C}}_{k}^{p}\right)}^{-1}+\frac{1}{N}\sum_{i=1}^{m}{\left(H_{k}^{i}\right)}^{T}{\left(C_{k}^{z,i}\right)}^{-1}H_{k}^{i}\right)\\ =N{\left({\bar{C}}_{k}^{p}\right)}^{-1}+\sum_{i=1}^{m}{\left(H_{k}^{i}\right)}^{T}{\left(C_{k}^{z,i}\right)}^{-1}H_{k}^{i}\\ ={\left(C_{k}^{p,c}\right)}^{-1}+\sum_{i=1}^{m}{\left(H_{k}^{i}\right)}^{T}{\left(C_{k}^{z,i}\right)}^{-1}H_{k}^{i}={\left(C_{k}^{e,c}\right)}^{-1}\;. \end{array}$$

Accordingly, ${\underline{\hat{x}}}_{k}^{e,d^{\prime }}={\underline{\hat{x}}}_{k}^{e,c}$ holds due to

$$\begin{array}{c} {\left(C_{k}^{e,d^{\prime }}\right)}^{-1}{\underline{\hat{x}}}_{k}^{e,d^{\prime }}=\sum_{i=1}^{m}{\left({\bar{C}}_{k}^{e}\right)}^{-1}{\underline{\bar{x}}}_{k}^{e,i}\;+\;\sum_{i=m+1}^{N}{\left({\bar{C}}_{k}^{p}\right)}^{-1}{\underline{\bar{x}}}_{k}^{p,i}\\ =\sum_{i=1}^{m}\left({\left({\bar{C}}_{k}^{p}\right)}^{-1}{\underline{\bar{x}}}_{k}^{p,i}+{\left(H_{k}^{i}\right)}^{T}{\left(C_{k}^{z,i}\right)}^{-1}{\underline{z}}_{k}^{i}\right)+\sum_{i=m+1}^{N}{\left({\bar{C}}_{k}^{p}\right)}^{-1}{\underline{\bar{x}}}_{k}^{p,i}\\ =\sum_{i=1}^{m}{\left({\bar{C}}_{k}^{p}\right)}^{-1}{\underline{\bar{x}}}_{k}^{p,i}+\sum_{i=m+1}^{N}{\left({\bar{C}}_{k}^{p}\right)}^{-1}{\underline{\bar{x}}}_{k}^{p,i}+\sum_{i=1}^{m}{\left(H_{k}^{i}\right)}^{T}{\left(C_{k}^{z,i}\right)}^{-1}{\underline{z}}_{k}^{i}\\ =N{\left({\bar{C}}_{k}^{p}\right)}^{-1}\frac{1}{N}\sum_{i=1}^{N}{\underline{\bar{x}}}_{k}^{p,i}+\sum_{i=1}^{m}{\left(H_{k}^{i}\right)}^{T}{\left(C_{k}^{z,i}\right)}^{-1}{\underline{z}}_{k}^{i}\\ ={\left(C_{k}^{p,c}\right)}^{-1}{\underline{\hat{x}}}_{k}^{p,c}+\sum_{i=1}^{m}{\left(H_{k}^{i}\right)}^{T}{\left(C_{k}^{z,i}\right)}^{-1}{\underline{z}}_{k}^{i}\\ ={\left(C_{k}^{e,c}\right)}^{-1}{\underline{\hat{x}}}_{k}^{e,c}={\left(C_{k}^{e,d^{\prime }}\right)}^{-1}{\underline{\hat{x}}}_{k}^{e,c}\;. \end{array}$$

 ☐

## Appendix B

In the following, relationship (20) is proven.

**Proof.** As a symmetric positive definite matrix, B can be written as a product $B=AA^{T}$, where A is a lower triangular matrix with positive diagonal entries, using the Cholesky decomposition. $A^{T}$ is then an upper triangular matrix with positive diagonal entries. As triangular matrices with positive diagonal entries, the matrices A and $A^{T}$ are invertible. We define $\underline{b}:=A^{-1}d_{k}^{i}$. We have then

$$\begin{array}{c} d_{k}^{i}{\left(d_{k}^{i}\right)}^{T}\leq B\Leftrightarrow B-d_{k}^{i}{\left(d_{k}^{i}\right)}^{T}\geq 0\Leftrightarrow AA^{T}-d_{k}^{i}{\left(d_{k}^{i}\right)}^{T}\geq 0\\ \Leftrightarrow A\left(I-A^{-1}d_{k}^{i}{\left(d_{k}^{i}\right)}^{T}{\left(A^{T}\right)}^{-1}\right)A^{T}\geq 0\Leftrightarrow I-A^{-1}d_{k}^{i}{\left(d_{k}^{i}\right)}^{T}{\left(A^{T}\right)}^{-1}\geq 0\\ \Leftrightarrow I-\underline{b}{\underline{b}}^{T}\geq 0\Leftrightarrow \underline{b}{\underline{b}}^{T}\leq I\;. \end{array}$$

We also have

$$\begin{array}{c} {\left(d_{k}^{i}\right)}^{T}B^{-1}d_{k}^{i}\leq 1\Leftrightarrow {\left(d_{k}^{i}\right)}^{T}{\left(A^{T}\right)}^{-1}A^{-1}d_{k}^{i}\leq 1\Leftrightarrow {\underline{b}}^{T}\underline{b}\leq 1\Leftrightarrow 1-{\underline{b}}^{T}\underline{b}\geq 0\;. \end{array}$$

We still have to show that

$$\begin{array}{c} I-\underline{b}{\underline{b}}^{T}\geq 0\Leftrightarrow 1-{\underline{b}}^{T}\underline{b}\geq 0\;. \end{array}$$

First, we will show “⇒”. From $I-\underline{b}{\underline{b}}^{T}\geq 0$ we have ${\underline{a}}^{T}\underline{a}-{\underline{a}}^{T}\underline{b}{\underline{b}}^{T}\underline{a}={\underline{a}}^{T}\left(I-\underline{b}{\underline{b}}^{T}\right)\underline{a}\geq 0\;\forall \underline{a}\neq \underline{0}$. With $\underline{a}=\underline{b}$ we have ${\underline{b}}^{T}\underline{b}-{\underline{b}}^{T}\underline{b}{\underline{b}}^{T}\underline{b}\geq 0$. Dividing by ${\underline{b}}^{T}\underline{b}$ gives $1-{\underline{b}}^{T}\underline{b}\geq 0$ . We will now show “⇐”. From ${\underline{b}}^{T}\underline{b}\leq 1$ we have ${\underline{a}}^{T}\underline{b}\leq ∥\underline{a}∥∥\underline{b}∥\leq ∥\underline{a}∥$ and ${\underline{b}}^{T}\underline{a}\leq ∥\underline{a}∥∥\underline{b}∥\leq ∥\underline{a}∥\;\forall \underline{a}$. We have then ${\underline{a}}^{T}\underline{b}{\underline{b}}^{T}\underline{a}\leq {\underline{a}}^{T}\underline{a}\;\forall \underline{a}$. It follows ${\underline{a}}^{T}\left(I-\underline{b}{\underline{b}}^{T}\right)\underline{a}={\underline{a}}^{T}\underline{a}-{\underline{a}}^{T}\underline{b}{\underline{b}}^{T}\underline{a}\geq 0\;\forall \underline{a}\neq \underline{0}$ and thus, $I-\underline{b}{\underline{b}}^{T}\geq 0$. ☐

## Appendix C

We will now show that the resulting estimate is consistent, i.e., relationship (22) holds.

**Proof.** We have

$$\begin{array}{c} {\underline{\hat{x}}}_{k}^{e,d^{\prime \prime }}={\underline{\hat{x}}}_{k}^{e,d^{\prime }}-C_{k}^{e,d^{\prime }}{\left({\bar{C}}_{k}^{p}\right)}^{-1}\sum_{i=m+1}^{N}{\tilde{d}}_{k}^{i}\;. \end{array}$$

The expected estimation error is then given by

(A1) $$\begin{array}{c} E\left(\left({\underline{x}}_{k}-{\underline{\hat{x}}}_{k}^{e,d^{\prime \prime }}\right){\left({\underline{x}}_{k}-{\underline{\hat{x}}}_{k}^{e,d^{\prime \prime }}\right)}^{T}\right)\\ =E\left(\left({\underline{x}}_{k}-{\underline{\hat{x}}}_{k}^{e,d^{\prime }}\right){\left({\underline{x}}_{k}-{\underline{\hat{x}}}_{k}^{e,d^{\prime }}\right)}^{T}\right)\\ -E\left(\left({\underline{x}}_{k}-{\underline{\hat{x}}}_{k}^{e,d^{\prime }}\right){\left(C_{k}^{e,d^{\prime }}{\left({\bar{C}}_{k}^{p}\right)}^{-1}\sum_{i=m+1}^{N}{\tilde{d}}_{k}^{i}\right)}^{T}\right)\\ -E\left(\left(C_{k}^{e,d^{\prime }}{\left({\bar{C}}_{k}^{p}\right)}^{-1}\sum_{i=m+1}^{N}{\tilde{d}}_{k}^{i}\right){\left({\underline{x}}_{k}-{\underline{\hat{x}}}_{k}^{e,d^{\prime }}\right)}^{T}\right)\\ +E(\left(C_{k}^{e,d^{\prime }}{\left({\bar{C}}_{k}^{p}\right)}^{-1}\sum_{i=m+1}^{N}{\tilde{d}}_{k}^{i}\right)\cdot \\ \;\;{\left(C_{k}^{e,d^{\prime }}{\left({\bar{C}}_{k}^{p}\right)}^{-1}\sum_{i=m+1}^{N}{\tilde{d}}_{k}^{i}\right)}^{T})\;. \end{array}$$

Due to the orthogonality principle [44] we have

$$\begin{array}{c} E\left(\left({\underline{x}}_{k}-{\underline{\hat{x}}}_{k}^{e,d^{\prime }}\right){\left(C_{k}^{e,d^{\prime }}{\left({\bar{C}}_{k}^{p}\right)}^{-1}\sum_{i=m+1}^{N}{\tilde{d}}_{k}^{i}\right)}^{T}\right)=0\;, \end{array}$$

and

$$\begin{array}{c} E\left(\left(C_{k}^{e,d^{\prime }}{\left({\bar{C}}_{k}^{p}\right)}^{-1}\sum_{i=m+1}^{N}{\tilde{d}}_{k}^{i}\right){\left({\underline{x}}_{k}-{\underline{\hat{x}}}_{k}^{e,d^{\prime }}\right)}^{T}\right)=0\;. \end{array}$$

We can now write (A1) as

$$\begin{array}{c} C_{k}^{e,d^{\prime }}+C_{k}^{e,d^{\prime }}{\left({\bar{C}}_{k}^{p}\right)}^{-1}E\left(\sum_{i=m+1}^{N}\sum_{j=m+1}^{N}{\tilde{d}}_{k}^{i}{\left({\tilde{d}}_{k}^{j}\right)}^{T}\right)\cdot \\ \;{\left({\bar{C}}_{k}^{p}\right)}^{-1}{\left(C_{k}^{e,d^{\prime }}\right)}^{T}\;. \end{array}$$

To complete the proof we still have to show that

$$\begin{array}{c} E\left(\sum_{i=m+1}^{N}\sum_{j=m+1}^{N}{\tilde{d}}_{k}^{i}{\left({\tilde{d}}_{k}^{j}\right)}^{T}\right)\leq {\left(N-m-l\right)}^{2}B\;. \end{array}$$

For i=j and i,j∈{m+1,…,N} we have

$$\begin{array}{c} {\tilde{d}}_{k}^{i}{\left({\tilde{d}}_{k}^{j}\right)}^{T}\leq B\;, \end{array}$$

since no communication was performed from sensor *i* to the center at time *k*. For i≠j and i,j∈{m+1,…,N}, we have

$$\begin{array}{c} 0\leq \left({\tilde{d}}_{k}^{i}-{\tilde{d}}_{k}^{j}\right){\left({\tilde{d}}_{k}^{i}-{\tilde{d}}_{k}^{j}\right)}^{T}\\ ={\tilde{d}}_{k}^{i}{\left({\tilde{d}}_{k}^{i}\right)}^{T}-{\tilde{d}}_{k}^{i}{\left({\tilde{d}}_{k}^{j}\right)}^{T}-{\tilde{d}}_{k}^{j}{\left({\tilde{d}}_{k}^{i}\right)}^{T}+{\tilde{d}}_{k}^{j}{\left({\tilde{d}}_{k}^{j}\right)}^{T}\;. \end{array}$$

We have then

$$\begin{array}{c} {\tilde{d}}_{k}^{i}{\left({\tilde{d}}_{k}^{j}\right)}^{T}+{\tilde{d}}_{k}^{j}{\left({\tilde{d}}_{k}^{i}\right)}^{T}\leq {\tilde{d}}_{k}^{i}{\left({\tilde{d}}_{k}^{i}\right)}^{T}+{\tilde{d}}_{k}^{j}{\left({\tilde{d}}_{k}^{j}\right)}^{T}\leq 2B\;. \end{array}$$

The number of summands different from zero in the sum $\sum_{i=m+1}^{N}\sum_{j=m+1}^{N}{\tilde{d}}_{k}^{i}{\left({\tilde{d}}_{k}^{j}\right)}^{T}$ is ${\left(N-m-l\right)}^{2}$. Thus, we have

$$\begin{array}{c} \sum_{i=m+1}^{N}\sum_{j=m+1}^{N}{\tilde{d}}_{k}^{i}{\left({\tilde{d}}_{k}^{j}\right)}^{T}\leq {\left(N-m-l\right)}^{2}B\;. \end{array}$$

It follows that

$$\begin{array}{c} E\left(\sum_{i=m+1}^{N}\sum_{j=m+1}^{N}d_{k}^{i}{\left(d_{k}^{j}\right)}^{T}\right)\leq {\left(N-m-l\right)}^{2}B\;. \end{array}$$

☐
